# Motives of Indian Nurses Moving to Germany in the Context of Skilled Labor Migration in Healthcare

**DOI:** 10.3390/nursrep16050178

**Published:** 2026-05-21

**Authors:** Matthias Pilz, Lydia Sterzenbach, Annabell Albertz

**Affiliations:** Chair of Business Education and International VET Research, University of Cologne, 50931 Cologne, Germany; lydia.sterzenbach@gmail.com (L.S.); annabell.albertz@uni-koeln.de (A.A.)

**Keywords:** migration, India, Germany, nurses, motives

## Abstract

**Background/Objectives:** Skilled labor migration of nurses from low- and middle-income to high-income countries is a growing global phenomenon. While Germany increasingly recruits internationally trained nurses to address severe nursing shortages, existing research has predominantly focused on post-migration experiences, leaving the pre-migration phase unexplored. Drawing on the case of Kerala, India, this study examines the motives driving Indian nurses to migrate to Germany during the preparatory phase of migration. **Methods:** The qualitative study draws on individual and group interviews with 22 Indian nurses from Kerala participating in a pre-migration preparatory course. Data were analyzed using qualitative content analysis, with Herzberg’s two-factor theory serving as a theoretical lens. **Results:** In total, the findings demonstrate that nurses’ decisions to migrate to Germany are shaped by a complex and interrelated set of motives encompassing personal fulfillment, socio-cultural aspirations and professional development. The intrinsic motivation to engage with a new language and culture emerged as a particularly salient finding, representing a dimension frequently overlooked in existing research. Applying Herzberg’s theory, migration decisions appear to result from an interplay of intrinsic motivators like career advancement and the perceived absence of fundamental extrinsic conditions such as adequate salary and social security in the country of origin. **Conclusions:** The findings have practical implications for practitioners, policymakers, employers and training providers. Preparatory programs could extend beyond language and professional training to include cultural preparation and realistic expectation management, while integration measures in Germany could be tailored to the actual needs and motives of internationally recruited nurses.

## 1. Introduction

Like many industrialized countries, Germany has faced an ongoing shortage of skilled labor for several years, particularly in healthcare [1]. This situation is expected to worsen in the upcoming years as demographic change leads to both an aging population and a decline in the native workforce, thereby further widening the gap in nursing staff. Projections estimate a shortfall of over 500,000 full-time nurses in Germany by 2030 [2]. To address this challenge, a multifaceted approach is required that fosters domestic labor potential and strengthens international recruitment through employment and training migration.

While migration from European countries still accounts for the majority of labor mobility, the demographic changes are increasingly shifting attention toward skilled labor migration from non-European and developing countries [1,3]. Germany has a longstanding history of recruiting foreign nursing personnel, dating back to the 1950s when Asian nurses were actively recruited for German hospitals. In recent decades, India has emerged as a key source country [4], which is reflected in numerous governmental and private Indo-German project activities and high numbers of program participants migrating to Germany as skilled workers [5,6,7].

Existing research on nurse migration has predominantly concentrated on the underlying push and pull factors of migration and the economic implications for the countries of origin, e.g., [8]. The majority of studies examine the experiences of nurses who have already migrated, often focusing on the challenges encountered in the destination countries [9,10]. This primary focus on experiences after migration leads to a considerable gap, as the motives, expectations, and perceptions of nursing staff before migration remain largely unexplored. However, a comprehensive understanding of the motives of nurses prior to their migration is essential for designing effective preparation and integration measures in both the country of origin and the destination country.

Against this backdrop, this study addresses this research gap by examining the migration motives of Indian nurses, with a particular focus on the southern state of Kerala. Kerala is recognized for its strong nursing training system and a longstanding tradition of international nurse migration [11,12], including to Germany [13,14,15]. Nurses from Kerala are in global demand due to their high-quality training and professional reputation [16,17]. Focusing on nurses who are at the final stage of preparing for migration to Germany, this study offers deeper insights into their individual motives for migration [18]. The investigation of this particular migratory phase enables a more precise comprehension of the nurses’ expectations and aspirations, which might otherwise be obscured subsequent to migration due to novel experiences and alterations in perspective. The central research question guiding this investigation is as follows: What motives guide nurses from India during the preparatory phase of migration in their decision to migrate to Germany as skilled workers? In this way, the study provides an empirically grounded contribution to a deeper understanding of the diverse motives underlying Indian nursing staff’s decision to migrate, with a specific focus on India as the country of origin and Germany as the destination country. Answering the research question is not only of academic interest but also has practical relevance: insights into the diverse and varied motives of Indian nurses can inform the design of preparatory programs in India such as language and cultural training, ensuring they are better tailored to the actual needs and wishes of nurses. In doing so, the findings can assist practitioners, policymakers, employers, and training providers in developing more targeted and sustainable migration strategies.

In order to address the research question, the presentation of the current state of research is outlined below. This is followed by an overview of the contextual background, theoretical framework and methodological approach. The final sections present and discuss the empirical findings.

## 2. State of Research

The international migration of nurses is a complex and multifaceted phenomenon shaped by an interplay of economic, political, social, professional, educational and personal factors [19]. Rather than being driven by a single cause, nurses’ decisions to migrate typically reflect a combination of structural challenges in their countries of origin and perceived opportunities abroad. Push factors include limited job opportunities, low wages, poor working conditions, and socio-economic disadvantage, while pull factors often involve higher salaries, better employment prospects, family ties and improved quality of life [20]. While the push–pull model offers a useful starting point, it has been criticized for oversimplifying migration decisions by reducing them to structural forces, thereby disregarding individual agency, cultural motivations, and context-specific dynamics [12].

Economic motives are among the most influential drivers of nurse migration. Significant wage disparities between sending and receiving countries serve as powerful incentives. For instance, Korean nurses report that German salaries are up to four times higher than in Korea [21]. These income differences allow nurses to support their families and improve their standard of living, even when adjusted for the higher cost of living in host countries [22,23]. However, economic incentives alone are not sufficient to fully explain migration decisions, including in the Indian context, where social, family, and professional aspirations play an equivalent role [24].

Beyond economic factors, political instability and systemic inequalities in sending countries also influence migration decisions. In Lebanon, political unrest and violence have prompted nurses to seek safety abroad [25], while Korean nurses have cited a desire for personal freedoms alongside career goals [26]. In India, caste-based discrimination and poor conditions in both public and private healthcare sectors act as push factors. The lack of fair treatment and limited access to secure, well-paid government positions lead many nurses to pursue work overseas [27,28]. This makes international migration in India an attractive and socially accepted pathway [11].

Social and professional aspirations likewise shape migration choices. Many nurses aim to secure a better future for their families through access to education and higher living standards [29]. Career development, skill enhancement and social recognition are additional motivating factors [22]. A study in four African countries found that nurses valued quality of life, career advancement and long-term opportunities as much as financial incentives [30]. In Nigeria, the absence of professional growth and high workloads contributed to a loss of professional identity, making migration a route to socio-economic empowerment [29]. Therefore, migration represents a means of both socio-economic and professional empowerment [31]. Notably, migration intentions often precede the nursing career itself. In countries like South Africa and India, individuals choose nursing as a strategic entry point into global labor markets rather than out of vocational interest [9]. Families may encourage this pathway, viewing the profession as a reliable means to achieve socio-economic mobility abroad [32]. This finding is interesting for the present study, as it suggests that the motives for migration may be rooted in personal and family life trajectories that developed before the actual migration process began. Therefore, the pre-migration phase is an issue that requires closer examination.

Despite strong motivations, the migration journey entails substantial challenges. The process of credential recognition and immigration can be complex and costly, and migrant nurses often rely on recruitment agencies, whose services vary in quality and transparency [33]. In the destination country, experiences with integration can be mixed: although internationalization of healthcare teams is often welcomed, nurses frequently report isolation and cultural adjustment difficulties [9]. Language proficiency is a critical barrier to both professional performance and social integration. Nurses face difficulties with medical terminology, cultural nuances and workplace communication, especially in countries where healthcare practices emphasize autonomy and flat hierarchies [34]. These challenges can affect confidence and psychological well-being. Targeted preparation programs and support networks have proven helpful in easing the transition for migrant nurses [35], and cultural and professional integration is more successful when these programs are tailored to the specific motives and needs of nurses [36].

Overall, existing research findings provide insights into the drivers of migration among nursing staff, yet some gaps in knowledge remain, including those relating to India and Germany. The pre-migration phase receives little attention in research, and cultural motives, such as the desire to acquire language skills or integrate into a new cultural context, are rarely highlighted. This study fills these gaps by examining the individual motives of Indian nurses who are actively preparing to migrate to Germany, thereby making a context-specific and temporally differentiated contribution to the existing literature.

## 3. Contextual Conditions

India offers a diverse array of nursing training programs, catering to a wide range of professional aspirations and educational levels ranging from diploma-level qualifications to advanced academic degrees [37,38]. The two programs most relevant to this study are briefly outlined below. The ‘General Nursing & Midwife Program’ is a three-and-a-half-year diploma course and takes place at hospital-affiliated nursing schools. Its primary objective is to prepare nursing personnel capable of delivering effective and competent care across a wide spectrum of healthcare settings. The program is designed to support both personal and professional development and to enable graduates to contribute to disease prevention, health promotion, rehabilitation and lifelong learning [39]. The ‘Bachelor of Science in Nursing’ is a four-year university-based undergraduate program. The program aims to develop professional nurses equipped to work in primary, secondary and tertiary healthcare environments. It prepares trainees not only for clinical practice but also for roles in nursing education and administration [38].

India’s long-standing tradition of nursing training is particularly prominent in the southern states. Kerala, in particular, has played a pivotal role in shaping the country’s nursing landscape. Together with Karnataka, Andhra Pradesh and Tamil Nadu, Kerala hosts more than half of India’s nursing institutions. This concentration is attributed to the region’s high levels of urbanization and well-developed hospital infrastructure. Kerala alone accounts for the largest number of registered nurses in the country [37,38,40]. The southern state’s training infrastructure and its strong tradition in nursing has also contributed to relevant patterns of international nurse migration, including to Germany. These migration pathways date back to the 1960s and 1970s, when Catholic and church-affiliated networks facilitated the recruitment of Indian nurses to German hospitals. This historical development laid the groundwork for institutionalized migration channels that still exist today, including formal bilateral cooperation programs and private recruitment initiatives specifically targeting nursing staff from Kerala for the German healthcare sector [7,15]. Given its long-standing tradition of both nursing training and international migration, Kerala is a particularly suitable study site for this research. The sample was therefore drawn from nurses in Kerala who were actively participating in a preparatory program for migration to Germany.

## 4. Theoretical Foundation

In the context of migration research, the aforementioned forces for migration, known as push and pull factors, compiled by [41], are frequently employed as a theoretical foundation, in the case of the migration of nurses [8,20,42,43]. However, given that the present study aims to explore the migration motives of Indian nurses in greater depth and thus goes beyond the mere analysis of push and pull factors, a specific strand of motivation theory is utilized as a theoretical basis. According to [44], motives are internal states that energize and direct behavior, thereby shaping individual tendencies and actions. Although motives are inherently subjective, they are often manifested in observable patterns of behavior. Motivation, derived from the concept of motive, can thus be conceptualized as an internal driving force that compels individuals to act in ways that fulfill their basic needs or desires [45].

Job satisfaction is a closely related concept, defined as a multidimensional construct encompassing individuals’ attitudes, perceptions and emotional responses to their work. Job satisfaction reflects the degree to which individuals find their job fulfilling or dissatisfying and is often regarded as a critical determinant of workplace performance, well-being and professional engagement [46,47]. Labor migration is intertwined with job satisfaction. The motivation for migration can be driven by the pursuit of enhanced job satisfaction and fulfillment, with both intrinsic and extrinsic factors playing a role [48,49]. A widely applied framework for understanding job-related satisfaction is Herzberg’s two-factor theory, which distinguishes between motivators and hygiene factors [50,51]. The motivators are intrinsic factors that increase job satisfaction, while the hygiene factors are extrinsic aspects that are intended to prevent dissatisfaction. The intrinsic and extrinsic factors are independent of each other [45]. Ref. [52] thus distinguishes between job satisfaction and no job satisfaction as well as between job dissatisfaction and no job dissatisfaction. Herzberg’s theory posits that motivators are the catalyst for job satisfaction and thus encourage employees to work hard and enjoy their work. Consequently, motivators should be integrated into daily working life to develop intrinsic motivation [45]. Motivators encompass the work itself, responsibility, performance, recognition, opportunities for promotion and personal growth. Hygiene factors, on the other hand, can lead to job dissatisfaction if they are lacking in a professional environment. These factors include, for example, company policy, supervision, job security and conditions, as well as salary, status and interpersonal relationships. Hygiene factors do not necessarily engender heightened motivation; nevertheless, their absence can give rise to dissatisfaction [45].

Herzberg’s two-factor theory has been applied and critically reflected upon in many contexts, e.g., [53,54]. The theory has also been discussed in the healthcare sector, particularly in developing countries, with the aim of understanding and enhancing the motivation of nursing staff. For instance, in [55] conducted in North Vietnam, health workers identified various motivating and demotivating factors. The study revealed that appreciation and support from superiors, colleagues and patients were identified as key motivators, highlighting the importance of performance recognition, respect, job security and personal growth. Furthermore, [56,57] have demonstrated the applicability of Herzberg’s two-factor theory to nursing staff in developing contexts.

Since Herzberg’s two-factor theory has thus far been applied mainly in the context of motivation among nurses, this study represents an attempt to explore the application of the theory to the context of migration. The aim is not to further develop or test the theory but to use it as a theoretical lens to explore which motivators and hygiene factors identified by Herzberg can explain nurses’ decisions to migrate to Germany and what conclusions can be drawn from this regarding preparations for migration.

## 5. Research Design

The objective of this study is to identify the motives underlying nurses’ decisions to migrate as skilled workers, with a specific focus on the pre-migration phase. Addressing a gap in research that has largely overlooked this period, the findings aim to inform targeted preparatory and integration measures aligned with the actual motives of internationally recruited nurses. To gain an in-depth understanding of these individual motives, a qualitative research design was adopted, employing open-ended, semi-structured interviews. Unlike quantitative approaches, which are better suited to measuring the prevalence or distribution of predefined variables, qualitative methods allow for the exploration of meaning, context and nuance, enabling participants to articulate their motives in their own words and on their own terms [58]. Moreover, the chosen methodological approach reflects the assumption that migration decisions are complex, deeply personal and shaped by individual experiences, subjective perceptions and socio-cultural contexts [see Section 2 above].

A semi-structured interview guide was developed to enable systematic data collection while retaining sufficient flexibility to capture nuanced individual perspectives. The development of the interview guide was informed by insights from evaluation research, studies on quality assurance in educational institutions and the design of intercultural training programs [59], as well as by existing research on nurse migration motives and Herzberg’s two-factor theory [52], which served as a theoretical lens for structuring the thematic areas of inquiry. The guide focused on key thematic areas, including motivations for migration, migration preparation including language acquisition and cultural adaptation, expectations regarding professional and everyday working life in the destination country and prior knowledge related to migration procedures and professional integration in Germany. Prior to the field research, a small pre-study was conducted to establish contact with local stakeholders in Kerala and to test and subsequently adapt the interview guideline. The guideline was based on open-ended questions to allow participants to speak freely about their perspectives. During the interviews, the guide was used flexibly and not as a rigid script. This allowed the conversation to follow the participants’ lines of thought while ensuring that all key topics were addressed. To enhance data quality and create an open conversational atmosphere, care was taken to establish a trusting and comfortable relationship between the interviewer and the interviewees.

The empirical phase of the study was conducted in Kerala at the end of May 2024. Data collection comprised both individual and group interviews conducted in English [60]. At the outset of the fieldwork, individual interviews were prioritized in order to provide participants with a protected setting in which they could articulate personal motives, expectations, and concerns in detail. During the research process, however, it became evident that group interviews constituted a valuable methodological complement. In the group setting, participants often displayed a more open and relaxed interactional dynamic, which facilitated collective reflection, mutual clarification of experiences, and the articulation of shared frames of reference related to migration aspirations. These dynamics can be partly understood against the backdrop of India’s collectivist cultural context, in which shared deliberation is an anchored social practice [61]. The combination of individual and group interviews was used in order to capture both individual motives and socially embedded perspectives. In total, twelve individual and four group interviews were conducted, with two to three participants in each group, resulting in 22 nurses being interviewed. Of the 22 participants, 19 were female and three male, with an average age of 30 years. Educational backgrounds varied, with the majority holding a Bachelor of Science in Nursing and a smaller number having completed the General Nursing and Midwifery program. The average length of professional experience was four years. At the time of the interviews, all participants had either completed a preparatory German language course and an intercultural training workshop or were in the process of preparing for the relevant examinations. Participant recruitment was carried out in cooperation with the local training administration. Through this organization, potential candidates were approached in person and informed of the opportunity to participate in the study. No formal exclusion criteria were established; however, a pragmatic requirement was that communication between the participants, and the interviewer had to be possible in English. Following the initial interviews, snowball sampling was additionally employed, as this approach proved particularly appropriate given the close professional networks among migration-preparing nurses in Kerala, allowing for access to further participants who met the study’s requirements and were at a comparable stage of migration preparation. Since most of the participants were young women, a young female interviewer of a similar age was specifically chosen to minimize potential power imbalances and create a comfortable atmosphere of mutual respect.

Adherence to ethical principles was a priority in the research process. All participants were verbally informed about the study’s objectives, procedure, and intended use prior to the start of the interview and gave their verbal consent to participate. It was explicitly noted that participation was voluntary and could be discontinued at any time without providing a reason and without consequences. The study complied with the university’s guidelines for the handling of research data and ethical principles.

Following data collection, the interview recordings were partially transcribed according to the transcription conventions proposed by [62]. Anonymity and confidentiality of participants were ensured through pseudonymization; all collected data were stored exclusively internally and is not accessible to third parties. The data were analyzed with MAXQDA 24 software using the method of qualitative content analysis, which allows for a structured yet flexible interpretation of qualitative material [62]. The analysis proceeded in several stages. In the initial phase, a deductive approach was applied, drawing on the theoretical framework and the interview guide to develop a preliminary category system. These categories were subsequently reviewed, refined and consolidated through inductive and iterative engagement with the empirical material.

This process resulted in the identification of three categories of migration motives: *personal fulfillment, socio-cultural aspirations and professional development*. Personal fulfillment refers to intrinsically motivated aspirations, encompassing the desire for individual growth, cultural curiosity, language acquisition, a sense of adventure and the pursuit of a self-determined life abroad. The following key example can be cited for this category: ‘I would like to learn new languages and settle abroad, building a new life with more opportunities for me.’ Socio-cultural aspirations capture motives rooted in family obligations, social security and the desire for an improved standard of living for oneself and one’s relatives. This category can be illustrated by the following example: ‘My family is everything. Because my family is poor, I can earn money in Germany and send it home to my children and my parents.’ Professional development encompasses motives related to career advancement, access to further education, improved working conditions and the pursuit of greater recognition of the nursing profession. For the third category the following key example can be given: ‘Pursue proper career advancement. I am interested in continuing my education.’ To enhance analytical rigor, the emerging category system was discussed in peer exchanges within the research team, allowing for critical reflection on category definitions and interpretations. The credibility of the study was supported by the systematic documentation of analytical decisions and the close linkage between interview excerpts, codes, and categories. The issue of transferability was addressed by means of a detailed description of the research context and sample characteristics. The dependability and confirmability of the research were strengthened through transparent documentation of the analytical procedure and reflexive engagement of the research team with the data [63]. In addition, the data was discussed and reflected upon with a partner from the local training administration in Kerala in order to reduce the risk of linguistic and cultural misinterpretation and to ensure a higher degree of cultural sensitivity. Nevertheless, it should be noted that the researchers’ position as European, external and academic professionals may have influenced research design, interview situation, data collection and interpretation, potentially introducing bias into the findings and limiting their transferability to other contexts or samples.

## 6. Results

The motive of *personal fulfillment* encompasses a curiosity about a new language and culture, a propensity for adventure and connections to acquaintances already living in the destination country. Many nurses reported that their decision to migrate stemmed from early life experiences: ‘From my childhood, I like to work in any of the European countries’ [T7]. Migration is seen as a valuable opportunity to engage in cultural exchange: ‘I want to mix up cultures, and I think the mix of different cultures makes sense’ [T13].

Language acquisition is central to this process. Although English is commonly spoken among the nurses and used in other migration destinations like the UK and the US, the effort to learn German signals a strong commitment to integration. This was evident across the majority of participants, as emphasized by the fact that many of the nurses already tried to learn German prior to their enrolment in the preparatory German language course on site in Kerala and were already familiar with basic German expressions [T14; T16; T17; T18]. The desire to explore German culture, including its nature, traditions and cuisine, was also emphasized in the interviews, though the depth and specificity of cultural interest varied across participants [T3; T21; T22].

Living abroad with family was frequently cited as a source of fulfillment [T3; T11; T14], alongside the ability to practice nursing more meaningfully. This is reflected in the narrative of a nurse [T14], who shared that she loves working with people, would like to expand her work beyond India, and has therefore decided to migrate to Germany. A strong sense of autonomy underpins the migration choice: ‘Finally, I have taken the decision to go abroad. I have chosen my passion, working as a nurse. Finally, I have decided to go to Germany. No other can motivate me. I am the motivator of my life’ [T20]. Confidence in the preparation course also strengthened the determination to migrate. Social networks further reinforce the migration decision. The presence of friends and acquaintances in Germany enhanced feelings of security and support, facilitating the decision-making process for many participants [T1; T2; T3; T6; T11; T12; T16; T18; T21; T22].

The motive of *socio-cultural aspirations* includes the pursuit of a higher standard of living, access to quality healthcare and greater personal security. Nurses expressed disillusionment with the limitations of India’s healthcare system, contrasting it with Germany’s comprehensive social services: ‘Germany is well-advanced. Not like here. I know that Germany will provide life insurance to each and every person. Here in India, we are facing problems without insurance. Therefore, we can’t go anywhere’ [T14].

Germany’s infrastructure and perceived quality of life were positively highlighted: ‘Germany is very well-maintained with infrastructure. It has a very high standard of living with a good healthcare system’ [T17]. Furthermore, Germans are described as ‘welcoming people’ [T3] who are friendly, helpful and open:

‘Germany has people, who are interested. They take care of me and my family very much and they provide me with good support. Germany has a good health system. […] I have studied about Germany on Google, no other source, but I know […] Germany is protecting and supporting people in Kerala also. So, I would like to work in Germany. That is my ambition.’ [T20]

The statement makes it clear that the German society is held in high regard and that the desire for security and support is strong. However, it should be noted that these descriptions largely reflect the nurses’ anticipatory perceptions and expectations prior to migration, drawing on secondhand sources, rather than verified personal experience.

Migration is additionally motivated by the desire to secure the family’s future. This includes access to free education for children and the potential for family reunification: ‘Also, in Germany they have free education for the children, and I can get my husband so I can settle down in Germany’ [T18]. For many nurses, improving the living conditions of relatives in India was a central motive for migrating: ‘Because my family is a very poor family. So, I can earn money for my parents and send it home’ [T10].

*Professional development* is also an essential motive for migration including three dimensions of career advancement and specialization, improved working conditions and salary. Migration is seen as a pathway to a more stable and fulfilling nursing career with better career opportunities, earning potential, higher recognition of the nursing profession and access to comprehensive further training opportunities, as the following statement underlines:

‘I want to set my career as a good advance, so I enquired the features in Germany. And I have noticed that a better career settlement is offered in Germany and […] there are other advancements Germany is providing me compared to India. I think foreign countries like Germany are much better to advance my career.’ [T16]

Germany is perceived as offering better employment conditions, access to specializations and opportunities for further education: ‘Germany is also providing many specialties to learn, so I can develop myself’ [T16] and ‘I can learn and work with advanced technology’ [T3].

Interviewees expressed a desire to pursue higher qualifications, including Master’s degrees and management positions: ‘I want to do a higher study, of course, getting a higher position in my profession, like a nursing supervisor’ [T18]. Regarding working conditions, improved work–life balance, regulated working hours, the possibility of part-time work and recognition of nursing work were especially valued:

‘Germany is providing me a good working time. In India, we are working long hours and we, the nurses, have many mentally and physically issues […] and we are not paid much. […] So when coming to Germany […] there is a working schedule, and they have work life balance. Enough time to concentrate on work and concentrate on health and family. It’s better for us to work in Germany.’ [T16]

Finally, concerning salary, although not the sole motivator, financial stability and the ability to support family members were seen as important benefits: ‘One of the most attractive things is the salary package they are providing in Germany according to the experience and efficiency of the quality of the provision of care’ [T16]. Higher wages were interpreted not just as economic gains but as signs of professional appreciation.

In total, the findings demonstrate that nurses’ decisions to migrate to Germany are shaped by a complex and interrelated set of personal, socio-cultural and professional motives. While analytically distinct, these dimensions mutually reinforce one another and together form a coherent motivational structure. Personal fulfillment emerges as a central motive, linking individual aspirations with structural and professional considerations.

## 7. Discussion

This study investigated the motives of Indian nurses in the initial phase of their skilled labor migration process to Germany. At the time of the interviews, the nurses had not yet migrated and thus had no direct experience of the destination country. Consequently, their perspectives are primarily theoretical, influenced by hopes, expectations and idealized images of life and work in Germany. This initial ‘dreamer’ stage is characterized by aspirations rather than realities [64], a factor that must be considered when interpreting the findings.

The motives identified range from personal and socio-cultural factors to professional aspirations. These findings align with existing research on nursing migration, which has documented similar motives such as the pursuit of improved living standards, also for the family, better working conditions, enhanced career opportunities and higher income [22,29]. Nonetheless, this study expands upon prior research in several ways. First, by explicitly focusing on the pre-migration phase, it captures motives and expectations before they are shaped or altered by actual migration experiences, a perspective that has been lacking in the literature to date. Second, the findings shed light on the specific migration pattern from India to Germany, which, despite its growing practical relevance, has received comparatively little attention in academic research. In this study, the desire to engage with a new culture and language was identified as a salient, albeit frequently disregarded, factor. This finding represents a valuable contribution, as existing studies rarely focus on cultural curiosity and the intrinsic motivation to learn a new language and tend to prioritize economic and structural factors of migration [20]. The acquisition of a new language can be regarded not only as a legal obligation in the context of migration but also as a substantial personal undertaking, necessitating a high degree of dedication to the destination country and the integration of the individual into both private and professional spheres. Furthermore, it is of particular importance for nursing staff to learn and be proficient in the language of the destination country, as their professional ethics require them to be in constant contact with patients [65]. The socio-cultural context of nursing in India further contextualizes these findings. As noted before, caste-based discrimination and limited access to secure, well-paid positions have historically acted as push factors, making international migration a socially accepted and even desirable pathway [66]. However, this recent trend with an increase in international employment opportunities and a shift in social norms, have contributed to a resurgence of interest in the nursing profession across religious and caste demographics [31,67]. While this structural backdrop was not prominently articulated in the interviews themselves, it likely forms part of the broader social environment in which the nurses’ migration aspirations are embedded. Overall, these findings suggest that the motives of nursing staff planning to migrate are diverse and multifaceted, underscoring the need for research approaches that go beyond economic factors and take into account personal, cultural, and interpersonal aspects of the migration decision.

The two-factor theory of [52] was used in this study as a theoretical framework. Since the theory has been applied primarily in the context of motivation, its application here opens up the possibility of exploring if and how it can be transferred to the context of motives of migration. The results suggest that this transfer can be fruitful: both intrinsic motivators and extrinsic hygiene factors appear to be relevant to the migration decisions of the nursing staff interviewed. Intrinsic factors such as the desire for professional development, recognition, meaningful nursing practice, and career advancement can be attributed to Herzberg’s motivators, which are associated with job satisfaction and intrinsic motivation. In the Indian context, where participants perceive career advancement and professional development as largely unattainable, it is precisely these motivators that seem to drive the search for better conditions abroad and are viewed as achievable through migration. At the same time, extrinsic factors such as salary, social security, interpersonal relationships, work–life balance, improved standard of living, access to healthcare, family well-being, and personal safety were frequently cited by participants. In Herzberg’s terminology, these would be considered hygiene factors: their absence in the home context appears to contribute to dissatisfaction and create an incentive for migration, while their expected presence in Germany is perceived more as a prerequisite for stability than as an actual source of motivation. This interpretation aligns with Herzberg’s original distinction, although it must be noted that the theory was not developed for the migration context and cannot be applied with absolute precision. Personal fulfillment connects these two dimensions by linking intrinsic elements such as autonomy, cultural curiosity, and self-actualization with socially embedded aspects such as language acquisition. Overall, the results suggest that Herzberg’s distinction between motivators and hygiene factors offers a helpful, albeit not fully transferable, theoretical framework for migration motives. The findings suggest that migration decisions are rarely monocausal but result from an interplay of intrinsic aspirations and the perceived absence of fundamental extrinsic conditions in the country of origin.

The interviewed nurses articulated a profound necessity for structured support throughout the migration process. Evidence from the interviews suggests that informational and practical support prior to arrival is perceived as highly relevant, particularly regarding uncertainty around administrative procedures, professional recognition and everyday life in Germany. This is consistent with findings in the literature suggesting that the implementation of preparation and support programs can improve satisfaction and reduce staff turnover [68,69]. This appears relevant for employers, managers, and colleagues, who should seek to promote intrinsic motivators while also actively addressing extrinsic factors of dissatisfaction in both the Indian and German contexts.

A recurrent theme in the interviews was the contrast between the nurses’ diversified training and responsibilities in India and their expectations of professional practice in Germany. Nursing training in India tends to be more diverse and includes extensive theoretical and practical components [15,38]. As a result, Indian nurses often assume a broad range of responsibilities, which can result in disappointment if these responsibilities are not mirrored in Germany. Managing these expectations is essential, not only for the nurses themselves but also for their German employers and colleagues. Awareness of the nurses’ qualifications and skills could help avoid underutilization and prevent workplace tensions [70]. In addition, host institutions could be encouraged to invest in intercultural training for their existing employees to create an inclusive work environment where internationally trained nurses feel valued and supported.

The nurses interviewed in this study had an average of four years of professional experience, a factor that should be taken into consideration when designing training pathways. This finding underscores the importance of acknowledging and leveraging existing qualifications and professional experience in the integration process. Recent studies have demonstrated that foreign nursing staff who undergo training in Germany often achieve superior integration into the corporate culture compared to those trained abroad [71]. Early exposure to the German healthcare system, whether through formal training or transitional programs, appears to facilitate cultural, professional and linguistic integration [72]. In light of the aforementioned context, a hybrid or modular training model in Germany could offer a viable solution, enabling migrants to obtain the necessary qualifications and language skills whilst also valuing and incorporating their prior learning [73].

## 8. Conclusions

Considering the growing shortage of qualified nurses in Germany and the increasing reliance on recruiting skilled workers from abroad, this study examined the motives for migration among 22 nurses in Kerala during their preparatory phase for migration to Germany. The analysis revealed that the motives are multifaceted and can be categorized into three overarching domains. The first motive, personal fulfillment, encompasses an interest in new cultures and languages, a desire for new experiences and challenges and the existence of social networks in the destination country. The second motive, which is of a socio-cultural nature, is primarily concerned with the desire for higher living standards and qualities, the securing of the family’s future and financial support for the family in the country of origin. Incentives for the third motive, professional development, include career opportunities and earning potential as well as the prospect of further training and specialization in the nursing profession. Using the two-factor theory by [52] as a theoretical lens, the results suggest that this approach provides a useful, albeit not entirely transferable, foundation for understanding the motives for migration: both intrinsic motivators and extrinsic hygiene factors appear to influence nurses’ decisions to migrate.

The findings of the study have practical relevance for policymakers, employers and hosting institutions. Migration motives can provide guidance for the development of targeted measures to attract and retain foreign skilled workers. Moreover, the results obtained can be utilized to enhance the preparedness and effectiveness of structured integration processes among employers [74]. Based on the research results, implications for the future design of training programs in the context of migration processes for nursing staff were derived, emphasizing the necessity for comprehensive and detailed preparation [75]. This preparation could integrate both cultural and professional aspects, for instance through hybrid or modular training models that combine language acquisition, intercultural competence and professional qualification, to facilitate a smooth and successful transition. Such holistic preparation of nurses can therefore contribute to successful integration in the destination country, and the insights gained may equally inform the design of similar programs in other countries and contexts.

Nevertheless, it is important to acknowledge the limitations of this study. Firstly, the study is limited to the perspective of 22 nurses in India, which limits the generalizability of the results. Furthermore, it is important to recognize the potential for sampling bias, as the nurses invited to participate were likely considered among the most competent, motivated, or promising candidates who had thoroughly deliberated on their migration decision. This purposive selection may have influenced the data by overrepresenting positive attitudes and underrepresenting critical or dissenting perspectives. Future research could therefore focus on other actors and experiences of migrant nurses, including after their arrival in Germany. In order to gain a more comprehensive understanding, a longitudinal study design could be a suitable approach, collecting data at multiple stages of the migration process, before departure, during transition, and after arrival in the destination country. It would additionally be worthwhile to investigate the long-term implications of migration on integration and job satisfaction in Germany and to explore whether nurses’ expectations are confirmed or disappointed over time, findings that could further test the applicability of Herzberg’s theory in the migration context. Furthermore, comparative research among nurses from other countries could yield valuable insights, which may assist in the optimization of the recruitment and integration of nursing staff from abroad, thereby contributing to the resolution of the shortage of skilled workers within the German healthcare system.

## Data Availability

Data is available on request from the authors.

## Public Involvement Statement

There was no public involvement in any aspect of this research.

## Guidelines and Standards Statement

This manuscript was drafted against the APA 7th guidelines for qualitative research.
